# Household Preparedness and Preferred Communication Channels in Public Health Emergencies: A Cross-Sectional Survey of Residents in an Asian Developed Urban City

**DOI:** 10.3390/ijerph15081598

**Published:** 2018-07-27

**Authors:** Greta Tam, Zhe Huang, Emily Ying Yang Chan

**Affiliations:** 1 Jockey Club School of Public Health and Primary Care, The Chinese University of Hong Kong, Hong Kong, China; gretatam@cuhk.edu.hk; 2 Collaborating Centre for Oxford University and CUHK for Disaster and Medical Humanitarian Response, The Chinese University of Hong Kong, Hong Kong, China; huangzhe@cuhk.edu.hk

**Keywords:** disaster, household preparedness, infectious diseases

## Abstract

Disaster awareness and household preparedness are crucial for reducing the negative effects of a disaster. This study aims to examine the citizens’ preparedness level in the event of a general disaster or outbreak of infectious disease and to identify suitable channels for community disease surveillance and risk communication. We used a stratified random design to conduct a digit-dialed telephone survey in Hong Kong during February 2014. Level of disaster preparedness was examined according to the possession of disaster kit items. Associations between socio-demographic factors and good household preparedness were assessed using multiple logistic regression models. Preferences for infectious disease surveillance were collected and analyzed. There were 1020 respondents. Over half of the respondents (59.2%) had good household preparedness. After adjustment, female respondents, having higher education and higher household income were significantly associated with good household preparedness. Television and telephone were the preferred channels to obtain and report infectious disease information, respectively. In conclusion, general and specific infectious-disease household preparedness levels in Hong Kong were generally good. Tailored preparedness programs targeted to specific communities are necessary for those lacking preparedness. Risk communication and public health surveillance should be conducted through television and telephone, respectively.

## 1. Introduction

Disaster awareness and household preparedness are crucial for reducing the negative effects of a disaster [1]. According to the Center for Disease Control and Prevention (CDC), in the United States of America, disaster awareness is associated with household preparedness, which includes possessing an emergency kit [2]. Globally, campaigns have emphasized the importance of disaster kits. For example, the Australian government has guidelines on emergency kits, updates of alerts and warnings, as well as carrying out disaster education through schools and ongoing research [3]. The American government holds a ‘Get10’ campaign that publicizes a disaster kit [4]. The Canadian government provides guidelines of household emergency kits and organized a national Emergency Preparedness Week annually to promote emergency preparedness through local events and media coverage [5,6]. In Nepal, organized training programs and guidelines are provided on the preparation of emergency kits and family emergency planning [7,8].

Hong Kong is the city most at risk of natural hazards in Asia, ranking third in the world. As one of the wettest cities within the Pacific Rim region [9], Hong Kong is prone to typhoons, floods, and fires. Also, Hong Kong has a history of infectious disease epidemics due to a dense population and close connection to mainland China [10,11]: Avian influenza A (H5N1) in 1997 and 2003, SARS epidemic in 2003, and swine influenza H1N1 in 2009 [12,13,14,15]. Frequent travelers from and to the Mainland increase the risk of transmission of such viruses such as human influenza A H7N9 and H5N6, causing significant morbidity and mortality [16]. 

The Hong Kong government has attempted to reduce the injuries and damages caused by natural hazards by implementing early hazard warnings and emergency planning, such as the weather warning system and storm protection plans [17,18]. Nevertheless, individual household preparedness is also necessary to build a bottom up disaster resilient community. Although disaster household preparedness guidelines have been issued through leaflets by the government [19], no general campaigns have been conducted to increase awareness, as evidenced by a study that showed Hong Kong citizens had low perceived susceptibility and awareness of disasters [10]. This might be because few have endured any physical harm or loss of personal property caused by disasters. Regarding infectious disease emergencies, the Hong Kong government has mass media materials on influenza, which includes TV and radio announcements, pamphlets, and booklets [20]. Despite this, a study showed that Hong Kong citizens had low anxiety level towards A/H7N9, misconceptions such as mixing up A/H7N9 and seasonal flu as well mistaking the transmission routes. They were also lacking in preventive practices [21]. 

Suitable channels for risk communication are critical in targeting health promotion, raising disaster awareness and preparedness. Major channels available for Hong Kong citizens to obtain information on household preparedness for diseases include television, internet, and telephone. For the preparation for an outbreak of infectious diseases, the Center for Health Protection of the Department of Health has given advice to the public (e.g., clean hands with alcohol-based hand rub and put on surgical masks when infectious disease is prevalent [22]) through television advertisements and their official website to promote personal hygiene and reduce the chances of an infectious disease outbreak through public health education. 

Our study aims to assess the level of household preparedness for general disaster and infectious disease outbreak and preferred communication channels during 2014 when the A/H7N9 outbreak occurred in Hong Kong, an Asian developed urban city facing the double risks of natural hazards and infectious disease epidemics. Household preparedness levels are assessed based on whether households have an adequate supply of necessary items in their disaster kit in preparation for natural hazards and infectious disease epidemics. We also assess the likelihood of each item being stocked. We investigate what sociodemographic factors are associated with good household preparedness and whether vulnerable populations have better household preparedness. We examine the channels preferred by citizens for risk communication, according to different socio-demographic groups and for community disease surveillance. In addition, we explore citizens’ expectations of the government in risk communication and their willingness to co-operate with the government in community disease surveillance.

## 2. Materials and Methods 

### 2.1. Study Design and Study Population

**Figure 1:**
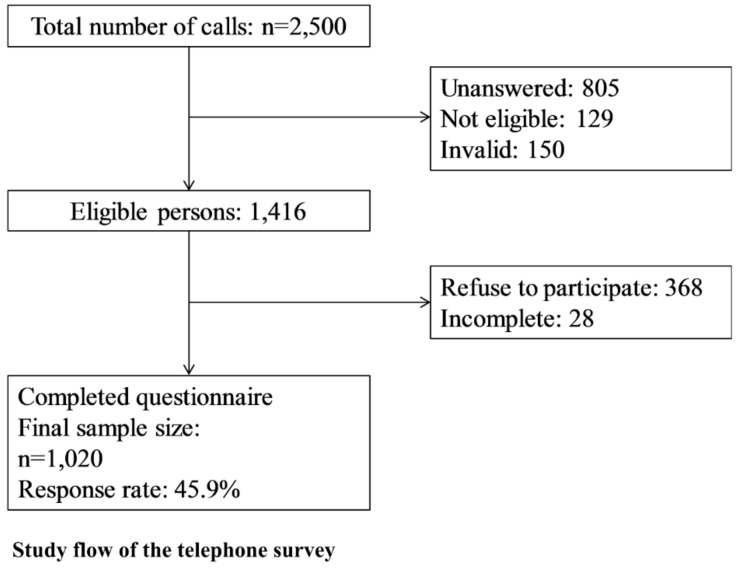
Study flow of the telephone survey.

A cross-sectional, randomized, population-based landline telephone survey was conducted on February 2014 in Hong Kong. A total of 2500 calls were made to the Cantonese-speaking population aged over 15 years who resided in Hong Kong including valid work or study visa holders. The flow of participant selection is shown in Figure 1.

Each interview lasted 15 to 25 min. A pilot study was conducted in January 2014 (*n* = 50) to test the practicability and validity of answers to the survey questionnaire. Wording and format were slightly modified based on the results of the pilot study.

The survey was completed when the second wave of the A/H7N9 epidemic occurred in Hong Kong. During this time, the total number of cases had risen to 320, compared to 135 in the first wave [23]. Confirmed case fatality rate was around 20% while the estimated symptomatic case fatality risk was lower [24]. Meanwhile, the infection rate of seasonal influenza was high in Hong Kong according to the Government Center for Health protection sentinel surveillance system [23]. The anxiety level of Hong Kong citizens was reported low in the first wave of the A/H7N9 outbreak [25]. 

### 2.2. Instrument

**Figure 2:**
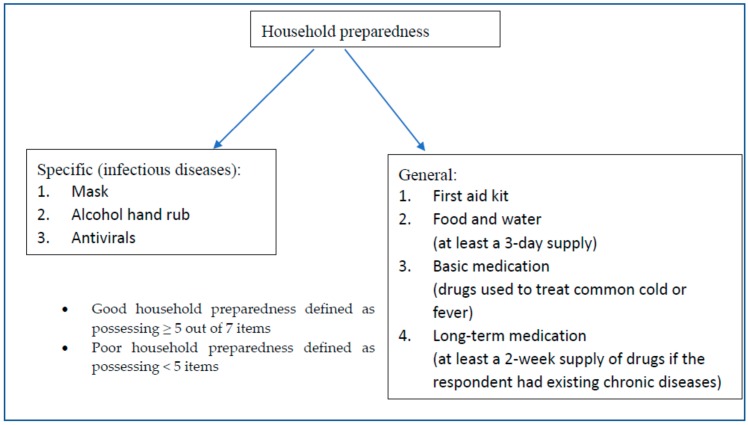
Definition of household preparedness levels and items.

A structured questionnaire was constructed and comprised of 78 closed-ended questions related to the information below:

- Socio-demographic and background information, including age, gender, district of residence, occupation and employment status, educational attainment, type and size of housing, and household income (21 questions). Vulnerable population referred to the elderly (>60 years old), those with respiratory or chronic diseases including asthma and hypertension and those who had flu in the past 2 weeks from the day of the interview.
- Knowledge, attitudes, and practices of preventive measures against A/H7N9 influenza infections (26 questions), reported elsewhere [21].
- Figure 2 summarizes the categories of household preparedness, the items for each category and the definition of household preparedness levels.

A cut-off of five items was used because two of the items may not necessarily be applicable to all citizens. As antivirals for influenza (e.g., Tamiflu) are prescription medicines, it would be unrealistic to expect all citizens to obtain this [26]. In addition, only households with members suffering from chronic disease would be expected to possess long-term medication. Thus, a household could still be termed as having good preparedness if they did not possess antivirals and long-term medication but possessed the remaining five essential items. Three of these essential items were derived from CDC recommendations and were chosen to represent a category: First aid kit represented “safety supplies”, food and water was itself a separate category, while basic medication represented “health supplies” [27]. These items were also included in similar surveys of disaster preparedness in Hong Kong [10,28] so that the findings were comparable. The remaining two essential items were specific to infectious diseases: masks and alcohol hand rubs were included as “cleaning hands with alcohol-based hand rubs and putting on surgical masks” were advice given to the Hong Kong public by the government [22].

Channel preference for obtaining and providing information to officials for surveillance and preference of internet use (total 30 questions) and a five-point Likert-type scale were used to ascertain the level of agreement or disagreement for the questions (from 1 to 5, 1 = strongly disagree, 2 = disagree, 3 = uncertain, 4 = agree, 5 = strongly agree).

### 2.3. Data Collection

Telephone numbers were generated randomly from the Hong Kong 2014 population telephone directory. Telephone interviews were conducted by trained interviewers from 6 p.m. to 10 p.m. on weekdays and 10 a.m. to 10 p.m. on weekends to prevent the under-representation of the employed population. Participants were chosen using the “last birthday method”, referring to the household member with the birthdate and month, ignoring year of birth, closest to the interview date [29,30]. Subjects were invited in proportion to the age, gender, and living district of the 2011 Hong Kong Population Census data. The sampling stopped when each stratum reached the limits. Selected participants were followed up by a maximum of four calls before classifying as unanswered. 

### 2.4. Statistical Analysis

Descriptive statistics of the household preparedness level and suitable channels for community disease surveillance and risk communication were presented. Likert-type scale results were collapsed to binary outcomes for analysis. Cut off point for questions with 5-point scales were defined as >3 and for questions with 4-point scale as >2. Univariate analysis was conducted by a logistic regression model to identify the association between the socio-demographic characteristics of respondents and good household preparedness. Subsequently, backward selection multivariable analysis determined factors that remained significantly associated with actual household preparedness. The association between a vulnerable population and good household preparedness was also examined. The results were presented in an adjusted odds ratio with 95% confidence intervals and *p*-values. All statistical analyses were conducted in R (R Core Development Team, version 3.0.3). 

## 3. Results

The final number of respondents included in the study was 1020, and the response rate was 45.9% (1020/2223). Table 1 shows the socio-demographic characteristics of the study population in comparison to the general population in Hong Kong in 2011.

### 3.1. Preparedness Level in General Disasters and Infectious Diseases Outbreaks

**Figure 3:**
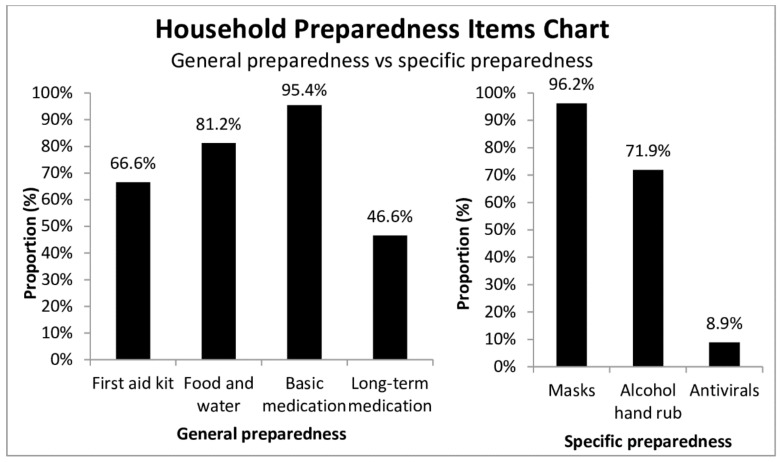
Proportion of respondents with household preparedness items (general and specific).

Most participants (59.2%) had good household preparedness (Figure S1), although only 3.4% of participants had a complete household preparedness kit. Although only 46.6% of general population possessed long-term medication, 157/206 (76.2%) respondents with chronic diseases possessed long-term medication (Figure 3). 

### 3.2. Characteristics of Respondents Lacking Household Preparedness

Univariate analysis of socio-demographics associated with good household preparedness was included in multivariable analysis. The remaining socio-demographic factors analyzed involved household-level characteristics: (Table 2).

We analyzed whether households with vulnerable members had better household preparedness, but found no significant association (Table S1). 

### 3.3. Suitable Channels for Community Disease Surveillance and Risk Communication

The preferred channels to obtain infectious disease information were from television (56%) and internet (16%). Meanwhile, smartphone/apps were one of the least popular sources. (Figure S2).

**Figure 4:**
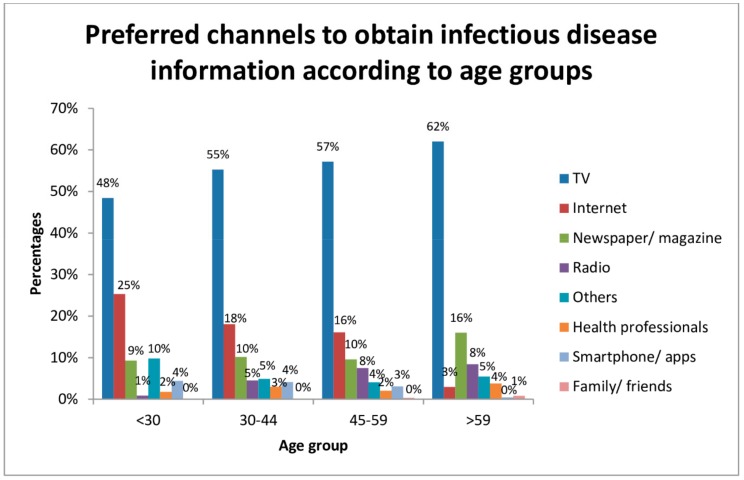
Preferred channels to obtain infectious disease information according to age groups.

Preferred channels according to demographics were analyzed (Figure 4: television was the most popular regardless of age, gender, occupation, education level, living quarters, household income, family size, and area of residence (not shown), while most respondents (75%) thought there was a need to have official indices that could easily communicate to the public the level of health risk of an infectious disease outbreak. Around 66% showed a willingness to cooperate with local officials for infectious disease data collection if needed. 

Preferred channels for providing health information to officials for surveillance purposes were telephone (61%) and online forms (48%) (Figure S3).

## 4. Discussion

We examined general and infectious disease-specific household preparedness levels and communication channel preferences. Most respondents had good household preparedness. Television and telephone were the preferred media for the Hong Kong public to obtain and report infectious disease information, respectively.

### 4.1. Household Preparedness Level

In this study, 59.2% of participants had good household preparedness (possessing at least five items. The kit items in our survey differed from the Security Bureau of Hong Kong guidelines [19], since infectious disease outbreaks were also considered. A similar study in Hong Kong examined the risk perception at the individual level and household level and assessed the household disaster preparedness level according to five measures among 1002 respondents: basic supplies, first aid kit, basic medication, non-perishable food, drinking water and fire extinguisher [10]. Half of the respondents reported being equipping with a first aid kit, 57.3% were equipped with non-perishable food and drinking water while 95.3% and 89.2% reported possessing basic and long-term medications, respectively. Our study was unique from the 2016 study of Chan et al. as infectious disease preparedness items (masks, antivirals, and alcohol hand rub) were incorporated in this study and *Chan* et al. only considered general disaster preparedness. A lower number of respondents possessed a first aid kit, food, and drinking water compared to our study. *Chan* et al.’s study was conducted two years before our study, and may indicate an improved general disaster preparedness over the years. Furthermore, similar findings were found in a previous study assessing families with young children in Hong Kong [28]. In Australia, a similar proportion of respondents possessed a first aid kit for preparedness against regular natural hazards such as bushfires, storms, and tropical cyclones [31]. In the USA and Canada, however, few had a good household preparedness level. In a USA study, only 8% had adequate food, water, and medication for 3-day survival in spite of significant frequency in hurricanes [32]. In Canada, few respondents possessed a 5-item disaster kit including a 3-day supply of canned food and water for each member of the household, a family evacuation plan, a portable battery-operated radio, a flashlight with functioning batteries, and home or apartment insurance for winter power-outages, fires, and medical emergencies [33]. The natural hazards anticipated in the USA and Canada include earthquakes, hurricanes, and tornadoes. The type of Hong Kong natural hazard differed, and correspondingly so did the necessary disaster kit items. Differing preparedness levels might be due to other countries’ perception that household preparedness was the government’s responsibility [33]. 

For infectious disease preparedness, few (8.9%) had antiviral medications in the present study. We were interested to see what proportion of the population possessed antivirals because although it is not currently a recommended practice, a study showed that antivirals for prophylaxis in the household might eliminate pandemic outbreaks [34]. Antivirals were important drug agents recommended by the WHO in promptly treating viral infections for high-risk individuals including seasonal influenza and preventing serious complications such as pneumonia [35]. They can be used as an alternative to vaccination. If vaccination cannot cover the circulating flu strain, such as A/H7N9, the chance of widespread transmission increases. Early detection and delivery of antivirals within 24 hours are crucial for reducing transmission and reducing complications [36]. Citizens might also be showing interest in obtaining antivirals because of anxiety over influenza outbreak: A study in Australia showed that 35% of respondents would store antivirals in preparation for pandemic influenza [37]. Although globally Tamiflu is only available as a prescription medicine [26], there have been reports of over the counter and online purchase of Tamiflu in Hong Kong [38] and other countries [39,40,41]. 8.9% of respondents may, therefore, reflect the eagerness of citizens to store antivirals at home. Further research is needed to explore how respondents obtain antivirals and the attitude of citizens towards the availability of antivirals.

Several determinants of health were associated with good household preparedness, consistent with previous studies [33,42,43,44,45]. In the current study, female respondents, having higher income and higher education level were associated with good household preparedness. Apart from differences in gender associated with good household preparedness [43,44], higher education and socioeconomic status (including higher income level) have been consistently associated with completion of disaster preparedness tasks such as storing food, water or first-aid supplies [33,42,45,46]. This demographic subgroup of individuals might possess greater self-efficacy, which has been shown to encourage disaster preparedness [47]. 

### 4.2. Preferred Channels in Different Countries

In Hong Kong, the penetration rate of licensed domestic free television service is 99%, which may explain the popularity of this channel for obtaining infectious disease information. USA citizens preferred obtaining health information on television news and newspapers [48]. Most in the UK also preferred television [49], as did Australians (31%). Only 13.9% of Australians preferred the internet, with 68.1% of respondents reporting home access [50]. Similar to Hong Kong, in the USA, internet popularity differs markedly between generations: 62% aged 18–29 preferred the internet compared to only 28% aged 60 or over [49]. This might be due to a divide of internet usage among age groups regarding skills including formal and operational skills [51]. Particularly low utilization of the internet in those aged over 65 in Hong Kong could explain why the internet was not preferred [52]. For information reporting, the telephone was preferred in the present study in spite of technology advancement. In the USA, internet was also not the most popular choice for reporting health information to healthcare providers [53]. The elderly seemed to prefer face-to-face interactions rather than using technology. In Australia, preferred channels of providing information for public health surveys varied across demographic characteristics. Younger individuals preferred online interviews while older ones preferred written questionnaires [54]. Only a few participants across age groups and sex preferred telephone questionnaire. In the instance of a pandemic, television and telephone should be feasible channels of communication. However, there are limitations in relation to natural hazards disabling such channels due to a lack of power or signal.

Our results show that Hong Kong citizens have relatively good household preparedness compared to other countries. Despite the relative self-sufficiency of citizens, many nevertheless hoped the government could do more in terms of risk communication for infectious disease. This could be because the risk of natural hazards is easily communicated through the Hong Kong Observatory’s weather warnings, using warning signals in their warning system, which are accompanied by suggested precautionary measures. In contrast, there are no official indices to indicate the risk of infection in a disease outbreak in Hong Kong. Citizens are exposed to media reports on the bi-yearly seasonal influenza and frequent reports on avian and swine flu waves. The information overload could cause pandemic fatigue and an inability to differentiate between influenza types. Official indices for infectious disease outbreaks, along with recommended precautionary measures specific to the disease, could be broadcast over television to simplify risk communication messages.

## 5. Limitations

This study is limited by methodological limitations of a cross-sectional telephone survey. Firstly, there may have been selection bias due to non-contact and non-response bias. Households that did not possess a land-based telephone service may be missed. The finding that most respondents preferred telephone for providing information to officials for surveillance may be influenced by selection bias. Nonetheless, the penetration rate of residential fixed line service in Hong Kong was 102.6% in November 2013, which implied that almost all households had at least one home-based telephone service in Hong Kong. To reach households that do not use landlines to communicate, alternative survey methods could be used; e.g., postal survey, online survey, or mobile phone survey. The sample population were more highly educated and had a higher household income than the general population. Thus, overestimation of the overall results may occur. Results may not be generalized, as other countries or cities have not experienced the same epidemiology of disasters. Reporting bias may be present due to self-reported data and missing data from non-respondents. Some factors that may be positively associated with participants’ household preparedness level including whether the participants ever receive any education or training for disaster preparedness before and or whether participants or their families or friends had negative experience related to disaster are missing for this survey. Finally, the consistency of the responses may be influenced by external factors during the survey period. Nevertheless, the field data collection was completed within a short period of two weeks to produce a consistent response. 

## 6. Conclusions

In conclusion, the general and specific infectious-disease household preparedness level in Hong Kong was generally good, with a small proportion of households possessing antivirals, despite over-the-counter unavailability. A tailored preparedness program to targeted communities is necessary for those lacking preparedness [31]. Educational program has been shown to increase both infectious disease and general disaster preparedness through talks and group discussions led by health promoters [55]. Since low-income households showed poorer preparedness, health campaigns should target them. Health campaigns could be held at public housing estates, as these households had poorer preparedness. Risk communication campaigns need to use the appropriate channels to increase effectiveness. As most citizens are willing to provide information to officials for surveillance, more frequent telephone surveys could be carried out during an infectious disease outbreak to strengthen surveillance. The results would also provide information for conducting tailored health campaigns. Health campaign efforts could focus on television, as this is by far the most popular channel across all demographic groups for obtaining information. There is also a demand for official indices, which would provide a direct and timely summary of the relevant health risk of infectious disease to the public.

## Figures and Tables

**Table 1:** Socio-demographic characteristics of respondents and the general population in Hong Kong 2011.

Demographics	Sample Population		Hong Kong Population 2011	Sample vs. Census *p*-Value ^a^
	*n*	%	%	
Age (*n* = 1020)				
15–24	143	14.0	14.0	0.99
25–44	348	34.1	35.5	
45–64	363	35.6	35.4	
≥65	166	16.3	15.1	
Gender (*n* = 1020)				
Male	461	45.2	46.0	1.00
Female	559	54.8	54.0	
Education (*n* = 1019)				
Primary education or below	138	13.5	22.7	0.18
Secondary education	517	50.7	50.0	
Post-secondary education (including diploma and certificate)	364	35.7	27.3	
Occupation (*n* = 1006)				
White collar	411	40.9	NA	
Blue collar	96	9.5	NA	
Housewife, retired or unemployed	393	39.1	NA	
Students	106	10.5	NA	
Area of residence (*n* = 1020)				
Hong Kong Island	185	18.1	18.0	1.00
Kowloon	308	30.2	29.8	
New Territories	527	51.7	52.2	
Marital status (*n* = 1018)				
Single	355	34.9	42.2	0.36
Married	663	65.1	57.8	
Household income (*n* = 969)				
<$10,000	135	13.9	23.8	0.30
$10,000–19,999	220	22.7	23.8	
$20,000–39,999	346	35.7	29.0	
≥$40,000	268	27.7	23.5	
Type of housing (*n* = 1017)				
Public housing	387	38.1	30.3	0.61 ^b^
Subsidized homeownership housing	160	15.7	15.9	
Private permanent housing	455	44.7	52.3	
Others	15	1.5	1.4	

^a^ Chi-square test was used to measure the overall difference in proportions between this survey and the 2011 Hong Kong Population Census data. *p*-Value < 0.05 indicates a significant difference. ^b^ Fisher-exact test *p*-value was used.

**Table 2:** Socio-demographic characteristics of respondents associated with good household preparedness.

Characteristics	Household Preparedness		^a^ COR (95% CI)	^b^*p*-Value	^c^ AOR (95% CI)	^b^*p*-Value
	Poor	Good				
	N (%)	N (%)				
***Respondents***						
***Gender***						
Male	214 (46.4)	247 (53.6)	1		1	
Female	202 (36.1)	357 (63.9)	1.53 (1.19, 1.97)	**<0.01**	1.63 (1.25, 2.21)	**<0.01**
***Occupation***						
White collar	156 (38.0)	255 (62.0)	1			
Blue collar	56 (58.3)	40 (41.7)	0.44 (0.28, 0.69)	**<0.01**		
Unemployed	162 (41.2)	231 (58.8)	0.87 (0.66, 1.16)	0.34		
Student	38 (35.8)	68 (64.2)	1.09 (0.70, 1.71)	0.69		
***Education***						
Primary education or below	75 (54.3)	63 (45.6)	1		1	
Secondary education	213 (41.2)	304 (58.8)	1.70 (1.16, 2.48)	**0.01**	1.68 (1.12, 2.53)	**0.01**
Post-secondary education (including diploma and certificate)	127 (34.9)	237 (65.1)	2.22 (1.49, 3.31)	**<0.01**	1.92 (1.21, 3.02)	**0.01**
***Household characteristics: Type of housing***						
Public housing	176 (45.5)	211 (54.5)	1			
Subsidized home ownership housing	71 (44.4)	89 (55.6)	1.05 (0.72, 1.51)	0.81		
Private permanent housing	164 (36.0)	291 (64.0)	1.48 (1.12, 1.95)	**0.01**		
***Household income***						
<$10,000	70 (51.9)	65 (48.1)	1		1	
$10,000–19,999	104 (47.3)	116 (52.7)	1.20 (0.78, 1.84)	0.40	1.12 (0.78, 1.73)	0.60
$20,000–39,999	140 (40.5)	206 (59.5)	1.58 (1.06, 2.36)	**0.02**	1.40 (0.93, 2.11)	0.11
≥$40,000	83 (31.0)	185 (69.0)	2.40 (1.57, 3.67)	**<0.0** **1**	2.01 (1.27, 3.17)	**<0.0** **1**
***Family size***						
1	38 (61.3)	24 (38.7)	1			
2	80 (40.8)	116 (59.2)	2.30 (1.28, 4.12)	**0.01**		
3–4	233 (39.9)	351 (60.1)	2.39 (1.39, 4.08)	**<0.01**		
≥5	65 (36.5)	113 (63.5)	2.75 (1.52, 4.99)	**<0.01**		

^a^ COR: Crude odds ratio; ^b^ Boldface indicates statistical significance; ^c^ AOR: Adjusted odds ratio; model was adjusted with gender, occupation, education, living quarters, household income, family size, and area of residence.

## Supplementary Materials

The following are available online at http://www.mdpi.com/1660-4601/15/8/1598/s1, Figure S1: The proportion of individuals against the number of household preparedness items (mask, alcohol hand rub, antivirals, first aid kit, food and water, basic medication, and long-term medication) at home. Figure S2: Preferred channels (TV, internet, newspaper/magazine, radio, others, health professionals, smartphone/apps, family/friends) to obtain infectious disease information. Figure S3: Preferred channels for providing health information to officials for surveillance Appendix: survey questionnaire. Table S1: Household preparedness of vulnerable population.
