# Supplementary material for: Household Preparedness and Preferred Communication Channels in Public Health Emergencies: A Cross-Sectional Survey of Residents in an Asian Developed Urban City
Source: Int J Environ Res Public Health. 2018 Jul 27;15(8):1598. doi: 10.3390/ijerph15081598 (PMC6121418; doi:10.3390/ijerph15081598)
Supplement: Supplementary file 1 [file ijerph-15-01598-s001.zip › IJERPHS1 Appendix.docx]

| **Knowledge, Attitudes, and Practices of Hong Kong population for influenza and pandemic preparedness of other infectious diseases after H7N9 avian influenza confirmed cases emerged in mainland China v2.10** | **Questionnaire No. ______________** |
| --- | --- |

Date: ________ Start Time: _________ Phone number of interviewees: _______________

Surveyor name: _____________________ Result double-checking: _______________________

Mr./Ms, Greetings!

I am an interviewer from the CUHK School of Public Health and Primary Care doing a telephone survey about infectious disease. May I ask if there is anyone older than 15 in this household? Who is the one with the most recent birthday? May I have him/her on the phone?

Greetings! I am an interviewer from the CUHK School of Public Health and Primary Care doing a telephone survey, aiming to investigate the perception and understanding of avian influenza and other infectious diseases by Hong Kong (HK) residents. The information collected can help us set more effective public health policies. The information you provide is for research use only and will not be disclosed. If you have any queries, please feel free to contact Prof. Emily Chan at 2252-8850. Thank you for your kind help!

0a) Do you agree to participate in this study?
1.Yes 2. No (End of interview. Thank you!)

0b) Do you agree that we record this conversation?
1.Yes (Start) 2. No (Start without recording)

**Part I: Background**

1. Do you have a Hong Kong Identity Card?

| 1. Yes - HK permanent resident |
| --- |
| 1. Yes - Non-permanent resident (e.g. domestic helper, holding working or study visa) 2. No- (End of interview. Thank you!) |

1. Gender: (if you have recognized, no need to ask) 1. Male 2. Female
2. What is your age?

| 1. 15-19 | 1. 20-24 | 1. 25-29 | 1. 30-34 |
| --- | --- | --- | --- |
| 1. 35-39 | 1. 40-44 | 1. 45-49 | 1. 50-54 |
| 1. 55-59 | 1. 60-64 | 1. 65-69 | 1. ≥70 |
| 999. Refuse to answer |  |  |  |

1. Do you think Hong Kong is susceptible to infectious diseases outbreaks?

| 1. No | 1. Yes | 888.Don’t know | 999.Refuse to answer |
| --- | --- | --- | --- |

1. Compared to other metropolitan cities (e.g. London, New York City, Tokyo), what do you think of Hong Kong’s overall preparedness for infectious disease outbreaks at city level?

| 1. HK is better than other metropolitans | 1. HK is worse than other metropolitan | 1. No difference |
| --- | --- | --- |
| 888. Don’t know | 999. Refuse to answer |  |

1. Have you caught cold /flu in the past 2 weeks?

| 1. No (ask 7) | 1. Yes (ask 6a & 6b) | 888.Don’t know | 999.Refuse to answer |
| --- | --- | --- | --- |

6a) Did you have fever at that time?

| 1. No | 1. Yes | 888.Don’t know | 999.Refuse to answer |
| --- | --- | --- | --- |

6b) How did you treat the disease?(If the respondent does not respond, then read options; After he\she answers, ask “are there more to it than that?”. May choose more than one choice)

1. Go to A&E 2. Goto Public GOPC (including DH/HA) 3. Goto Private GP

4. Goto Chinese Medical doctor 5. Self medication (Western) 6. Self medication (Chinese medicine) 7. Do Nothing 8 Others:_______________

| 888.Don’t know | 999.Refuse to answer |
| --- | --- |

1. Which weather do you think is easier for transmission of infectious diseases? Cold or hot weather? (Read out the answers)

1. Cold weather 2. Hot weather 3. Similar 888. Don’t know 999. Refuse to answer

1. Do you regard the indoor air pollution in your house as serious?

1. Worse than ordinary family 2. No difference 3. Better than ordinary family

888. Don’t know 999. Refuse to answer

1. Do you think H7N9 is the seasonal flu of this year?

1. No 2. Yes 3. Not clear 888. Don’t know 999. Refuse to answer

**Part II：Risk Perception of H7N9 Avian Influenza**

| *Please give a score from 1-5 of the following questions:* | | | | | | | | |  | Don’t know | Refuse |
| --- | --- | --- | --- | --- | --- | --- | --- | --- | --- | --- | --- |
| 1. Will H7N9 avian influenza adopt human to human transmission this year (If already adopted, code=6)? | *No chance at all* | | *1* | *2* | *3* | *4* | *5* | | *Very likely* | 888 | 999 |
| 1. You have very high chance to be infected by H7N9 avian influenza virus this year (If already infected, code=6)? | *Totally disagree* | *1* | *2* | *3* | *4* | *5* | | *Totally agree* | | 888 | 999 |
| 1. In what degree H7N9 avian influenza will affect your health status？ | *No impact at all* | *1* | *2* | *3* | *4* | *5* | | *Very high impact* | | 888 | 999 |
| 1. In what degree H7N9 avian influenza will affect your economic status？ | *No impact at all* | *1* | *2* | *3* | *4* | *5* | | *Very high impact* | | 888 | 999 |
| 1. In what degree H7N9 avian influenza will affect the whole HK society? | *No impact at all* | *1* | *2* | *3* | *4* | *5* | | *Very high impact* | | 888 | 999 |
| 1. The spread of H7N9 avian influenza can be prevented at government policy level? | *Totally disagree* | *1* | *2* | *3* | *4* | *5* | | *Totally agree* | | 888 | 999 |
| 1. The spread of H7N9 avian influenza can be prevented at household and individual level? | *Totally disagree* | *1* | *2* | *3* | *4* | *5* | | *Totally agree* | | 888 | 999 |
| 1. You have sufficient knowledge to manage the risks that H7N9 avian influenza brings to your health & security? | *Totally insufficient* | *1* | *2* | *3* | *4* | *5* | | *Very sufficient* | | 888 | 999 |
| 1. IClimate change will make influenza viruses mutate easier | *Totally disagree* | *1* | *2* | *3* | *4* | *5* | | *Totally agree* | | 888 | 999 |
| 1. Climate change will make people get infectious diseases easier | *Totally disagree* | *1* | *2* | *3* | *4* | *5* | | *Totally agree* | | 888 | 999 |
| 1. Indoor air pollution will make you more susceptible to infectious diseases | *Totally disagree* | *1* | *2* | *3* | *4* | *5* | | *Totally agree* | | 888 | 999 |
| 1. Outdoor air pollution will make you more susceptible to infectious diseases | *Totally disagree* | *1* | *2* | *3* | *4* | *5* | | *Totally agree* | | 888 | 999 |

**Part 3：Knowledge of H7N9 Avian Influenza**

|  |  |
| --- | --- |

1. Where do you usually obtain infectious diseases related information the most? (Choose only 1 answer)

| 1. TV | 1. Radio | | 1. Internet | 1. Newspaper/Magazine | |
| --- | --- | --- | --- | --- | --- |
| 1. Family/ friends | 1. Health professionals | | 1. Smart phone platforms / Apps | | |
| 1. Others (please specify) _________________ | 888. Don’t know | 999. Refuse to answer | | |  |

1. Where do you like to obtain infectious diseases related information the most? (Choose only 1 answer)

| 1. TV | 1. Radio | | 1. Internet | 1. Newspaper/Magazine | |
| --- | --- | --- | --- | --- | --- |
| 1. Family/ friends | 1. Health professionals | | 1. Smart phone platforms / Apps | | |
| 1. Others (please specify) _________________ | 888. Don’t know | 999. Refuse to answer | | |  |

1. Do you think H7N9 avian influenza can be spread by? (read out item a-g)

|  |  | Yes | No | Don’t know | Refuse |
| --- | --- | --- | --- | --- | --- |
| a. | Droplets by people | 1 | 2 | 888 | 999 |
| b. | Air borne | 1 | 2 | 888 | 999 |
| c. | Direct hand contact | 1 | 2 | 888 | 999 |
| d. | Indirect hand contact (e.g. via doorhandle) | 1 | 2 | 888 | 999 |
| e. | Faecal-Oral by people | 1 | 2 | 888 | 999 |
| f. | Animals | 1 | 2 | 888 | 999 |
| g. | insects | 1 | 2 | 888 | 999 |

1. The infectivity of the illnesses below is (from 1-5, 1=very low infectivity, 5=very high infectivity)

a. Severe Acute Respiratory Syndrome (SARS) 1 2 3 4 5 888 Don’t know 999 Refuse

b. H7N9 avian influenza 1 2 3 4 5 888 Don’t know 999 Refuse

c. H1N1 swine influenza 1 2 3 4 5 888 Don’t know 999 Refuse

d. Seasonal influenza 1 2 3 4 5 888 Don’t know 999 Refuse

e. Pneumococcal infection 1 2 3 4 5 888 Don’t know 999 Refuse

1. The severity of the illnesses below is (from 1-5, 1=very low severity, 5=very high severity)

a. Severe Acute Respiratory Syndrome (SARS) 1 2 3 4 5 888 Don’t know 999 Refuse

b. H7N9 avian influenza 1 2 3 4 5 888 Don’t know 999 Refuse

c. H1N1 swine influenza 1 2 3 4 5 888 Don’t know 999 Refuse

d. Seasonal influenza 1 2 3 4 5 888 Don’t know 999 Refuse

e. Pneumococcal infection 1 2 3 4 5 888 Don’t know 999 Refuse

1. In your opinion, the protection of influenza vaccine can last for?

1. Less than one year 2.One year 3 .Two years 4. Three years 5. More than 3 years 5. Lifelong

888. Don’t know 999. Refuse

| Part 4：Infectious Diseases Prevention and Practice   1. Do you have the habit of regular flu vaccines injection?   1 Never (ask 28a, and then ask 29) 2 sometimes but not regular (ask 28a, and then ask 29)  3.Yes (ask28b) 888 Don’t know 999 Refuse  28a) Why not? (no need to read out, only choose one item)  1. No time 2. Expensive 3. Afraid of adverse effects 4. Still will get infected after vaccination 5. Not necessary 6. Others: _________ 888. Don’t know 999 Refuse    28b) Why? (no need to read out, only choose one item)  1. Protect myself 2. Protect others 3. Necessary for work 4. Have subsidies (Government/Company) 5. Others: _________ 888. Don’t know 999 Refuse     1. Have you received seasonal flu vaccines in the past 6 months (the one in 2013-2014)?   1 No 2 Yes 888 Don’t know 999 Refuse   1. Will you take seasonal flu vaccines in future?   1 Probably not 2 half-half 3 Probably yes 888 Don’t know 999 Refuse   1. Do you think seasonal flu vaccination can protect you from H7N9 infections?   1 No 2 Yes 888 Don’t know 999 Refuse   1. If H7N9 protection is included in seasonal flu vaccine in future; will you take seasonal flu vaccine?   1 Probably not 2 half-half 3 Probably yes 888 Don’t know 999 Refuse   1. If there is a H7N9 vaccine in the future, will you receive the injection?   1 No (ask 33a, and then ask 34) 2 Yes (ask 33a, and then ask 34) 888 Don’t know (ask 34)  999 Refuse  33a) Why not? (no need to read out, only choose one item)  1. No time 2. Expensive 3. Afraid of adverse effects 4. Still will get infected after vaccination 5. Not necessary 6. Others: _________ 888. Don’t know 999 Refuse    33b) Why? (no need to read out, only choose one item)  1. Protect myself 2. Protect others 3. Necessary for work 4. Have subsidies (Government/Company) 5. Others: _________ 888. Don’t know 999 Refuse |
| --- |
|  |

| 1. Is your household always equipped with the following items? | No | Yes | Don’t know | Refuse |
| --- | --- | --- | --- | --- |
| a. Masks | 1 | 2 | 888 | 999 |
| b. Alcohol hand rub | 1 | 2 | 888 | 999 |
| c. First aid kit | 1 | 2 | 888 | 999 |
| d. Extra storage of food and water to sustain for at least 24 hours | 1 | 2 | 888 | 999 |
| e. Basic medication (e.g. pain killer, anti-pyretic) | 1 | 2 | 888 | 999 |
| f. Antivirals for influenza (eg. Tamiflu) | 1 | 2 | 888 | 999 |
| g. Long-term medication (enough for 1 week) | 1 | 2 | 888 | 999 |

1. Do you wash your hands before eating or after toilet?(If the respondent does not ask, no need to read the content bracketed)

①Always (>9/10 times) ②Usually (6-9/10 times) ③Sometimes (1-5/10 times) ④Never 888.Don’t know 999. Refuse

1. Do you use liquid soap when washing your hands? (If the respondent does not ask, no need to read the content bracketed)

①Always (>9/10 times) ②Usually (6-9/10 times) ③Sometimes (1-5/10 times) ④Never 888.Don’t know 999. Refuse

1. Do you use serving utensils when dining with others?(Both eating at home and outside)

①Always ②Usually ③Sometimes ④Never 888.Don’t know 999.Refuse

1. Do you bring your own utensils when dining out?

①Always ②Usually ③Sometimes ④Never 888.Don’t know 999.Refuse

1. Did you wear face mask and for how long during the most recent cold and flu incidence?

1=Always (more than 10 hours per day, excluding bed time)

2=Often (6-10 hours per day, excluding bed time)

3=Sometimes (less than 6 hours per day, excluding bed time)

4=Never

888=Don’t know

999=Refuse

1. Will you avoid going to public places or using public transport?

1 Completely avoid 2 Probably avoid 3 Sometimes avoid 4 Not avoid

888 Don’t know 999 Refuse

1. Will you avoid contacting with live poultry?

1 Completely avoid 2 Probably avoid 3 Sometimes avoid 4 Not avoid 888 Don’t know 999 Refuse

1. Will you avoid eating poultry?

1 Completely avoid 2 Probably avoid 3 Sometimes avoid 4 Not avoid 888 Don’t know 999 Refuse

1. Will you avoid going to the places having H7N9 confirmed cases? (eg. mainland China)

1 Completely avoid 2 Probably avoid 3 Sometimes avoid 4 Not avoid 888 Don’t know 999 Refuse

| 1. Do you think the following action can prevent H7N9 infections? | No | Yes | Don’t know | Refuse |
| --- | --- | --- | --- | --- |
| a. wash hands more often | 1 | 2 | 888 | 999 |
| b. use soap to wash hands more often | 1 | 2 | 888 | 999 |
| c. do not share utensils more often | 1 | 2 | 888 | 999 |
| d. wear mask more often when sick | 1 | 2 | 888 | 999 |
| e. bring our utensils | 1 | 2 | 888 | 999 |
| f. avoid go to public places and use public transport | 1 | 2 | 888 | 999 |
| g. avoid contacting with live poultry | 1 | 2 | 888 | 999 |
| h. avoid eating live poultry | 1 | 2 | 888 | 999 |
| i. avoid going the places having H7N9 confirmed cases | 1 | 2 | 888 | 999 |

Part 5：Surveillance when confronting a H7N9

1. If local officials or Universities would like to collect your health information for disease surveillance purposes regularly, are you willing to share these information?

| 1. No | 2. Yes | 888. Don’t know | 999. Refuse |
| --- | --- | --- | --- |

1. If you must report your health information to local officials, which method(s) would you like to use? (Read out the answer, can choose more than one category)

1. Telephone 2. Email 3. Online forms 4. Smart phone apps 5. Face to face interview

6. Mail 7. By hand in to relevant department 8. others: ________________________

1. Which area will you consider if you are going to assess your risk of H7N9 avian influenza infection? (Read out every answer, can choose more than one item)

1. How many people get infected 2. How frequent you contact with others 3. Your nature of work

4. The sanitation status of your living environment 5. Your health status 6. Health status of surrounding people 7. Others: _________________

1. What do you want to know more about H7N9 avian influenza (Read out every answer, can choose more than one item)?
   1. How to protect my family from being infected with this virus
   2. What is the current epidemic situation
   3. Can the virus transmit from person to person
   4. How can we get reliable information on the disease
   5. How to conduct home disinfection
   6. What kind of food can increase resistance of body to the virus
   7. Is the vaccination available? Is it safe
   8. How to do if I suspect I am infected with H7N9
   9. How to visit hospital
   10. Is there any effective drug treatment
   11. What preparatory work has been done by health department

888. Don’t know 999. Refuse

1. Do you think we need some sort of official guidelines or indices (eg. air quality、UV index etc) to help people in HK assess the risk infectious diseases infection?

| 1. No need | 2. Need | 888. Don’t know | 999. Refuse |
| --- | --- | --- | --- |

**Part 6：Anxiety level**

Related to infectious disease risk, right now

1. I feel calm 1. Not at all 2. Somewhat 3. Moderately so 4. Very much so 888. Don’t know 999. Refuse
2. I feel tense 1. Not at all 2. Somewhat 3. Moderately so 4. Very much so 888. Don’t know 999. Refuse
3. I feel upset 1. Not at all 2. Somewhat 3. Moderately so 4. Very much so 888. Don’t know 999. Refuse
4. I feel relaxed 1. Not at all 2. Somewhat 3. Moderately so 4. Very much so 888. Don’t know 999. Refuse
5. I feel content 1. Not at all 2. Somewhat 3. Moderately so 4. Very much so 888. Don’t know 999. Refuse
6. I am worried 1. Not at all 2. Somewhat 3. Moderately so 4. Very much so 888. Don’t know 999. Refuse

**Part 7：Supplementary information**

1. What is your ethnicity?

| 1. Chinese(Chinese origin, including all nationalities) | 1. Others (please specify) _________ | 888.Don’t know | 999. Refuse to answer |
| --- | --- | --- | --- |

1. What is your marital status?

| 1. Never married | 1. Now married | 1. Widowed | 1. Divorced |  |
| --- | --- | --- | --- | --- |
| 1. Separated | 888.Don’t know | 999.Refuse to answer |  | |

1. What is your highest education level obtained?

| 1. No schooling/pre-primary | 1. Primary | 1. Lower secondary | 1. Form 5 graduate |
| --- | --- | --- | --- |
| 1. Upper secondary \Advanced level | 1. Diploma/certificate | 1. Higher diploma | |
| 1. Sub-degree course | 1. Degree | 1. Master or above | |
| 11. Others (please specify) _____________________ | 888.Don’t know | 999.Refuse to answer |  |

1. What is your occupation?

| 1. Managers and administrators | | 1. Professionals | 1. Associate professionals | 1. Clerks |
| --- | --- | --- | --- | --- |
| 1. Service workers and shop sales workers | | 1. Craft and related workers | 1. Plant and machine operators and assemblers | 1. Elementary occupations |
| 1. Skilled agricultural and fishery workers | | 1. Housewives | 1. Student | 1. Retired   (ask 61) |
| 1. Unemployed   (ask 61) | 1. Others (please specify) ______________ | | 888.Don’t know | 999.Refuse to answer |

1. Do you consider your occupation is with high risk for infectious diseases infections?

(If the answer for question 59 is 10. Housewives, ask

“being a housewife, do you consider you are with high risk for infectious diseases infections?”

If the answer for question 59 is 11. Student, ask

“being a student, do you consider you are with high risk for infectious diseases infections?”)

| 1. No | 1. Yes | 888.Don’t know | 999.Refuse to answer |
| --- | --- | --- | --- |

1. Have you ever received Tetanus vaccines?(including all kinds of tetanus vaccines)

1 No (ask 61a then 62) 2 Yes (ask 61b then 62) 3 Cannot remember 888 Don’t know 999 Refuse

60a) Why not? 1.No time 2. Expensive 3. Afraid of adverse reaction 4. Still will get infected after shot 5. Not necessary 6. Others: __________ 888 Don’t know 999 Refuse

60b) Why? 1. Protect myself 2. Protect others 3. Necessary for work 4. After injury 5. Have subsidies (Government/Company) 6. Others: _________ 888. Don’t know 999 Refuse

1. What is your religion? (Don’t need to read out, can only choose 1)

| - 1. No religious belief | - 1. Christian | - 1. Catholic | - 1. Buddhist |
| --- | --- | --- | --- |
| - 1. Taoist | - 1. Muslim | - 1. Worship Chinese Gods \ ancestors at home   2. Others (please specify) __________________ | |
| 888.Don’t know | 999.Refuse to answer |  |  |

1. Will you burn incense regularly at your home? (not including aroma)

| 1. No(ask 64) | 1. Yes(including sometimes)   (ask 63a) | 888.Don’t know  (ask 64) | 999.Refuse to answer  (ask 64) |
| --- | --- | --- | --- |

62a) Is it traditional incense or electric incense?

1. Traditional incense 2. Electric incense 3. Both 888. Don’t know 999. Refuse to answer

1. Do you think burn incense at home is bad for health?

1. No idea 2. Is bad for health 3. Is not bad for health

1. Do you suffer from any chronic disease?

| 1. No(to Q.66) | 1. Yes (answer Q.65a&b) | 888.Don’t know (to Q.66) | 999.Refuse to answer (to Q.66) |
| --- | --- | --- | --- |

65a) What kind of chronic disease are you suffering from?

(Don’t need to read out, can choose more than 1)

| 1. Diabetes | 1. Hypertension | 1. Cardiovascular diseases | 1. Respiratory diseases |
| --- | --- | --- | --- |
| 1. Gastrointestinal diseases | 1. Others chronic conditions (eg. thyroid, joint pain etc) | 888.Don’t know | 999.Refuse to answer |

65b) Do you need to receive long term treatment?

| 1.No | 2.Yes | 888.Don’t know | 999.Refuse to answer |
| --- | --- | --- | --- |

1. Do any of your family members suffer from respiratory disease?(e.g. asthma, sensitive tracheal, chronic bronchitis)

| 1.No | 2.Yes | 888.Don’t know | 999.Refuse to answer |
| --- | --- | --- | --- |
|  |  |  |  |
|  |  |  |  |

1. Do any of your family members smoke at home, including you?

| 1.No | 2.Yes | 888.Don’t know | 999.Refuse to answer |
| --- | --- | --- | --- |

1. What is the type of your living quarters, including you?

| 1. Public housing | 1. House Ownership Scheme | 1. Private estates |
| --- | --- | --- |
| 1. Wooden house/temporary shelter | 1. Private house (village house or mansions) | 1. Sublet rooms |
| 1. Cubicle or bed-space | 888. Don’t know | 999. Refuse to answer |
| 1. Others (please specify) ___________ | | |

1. Including you, how many household members (including domestic helper) living together in your flat? __________ people 888. Don’t know 999. Refuse to answer
2. (if Q69=1, then ask Q71)Including you, are there any member in your household who is under 15 years old or older than 59 years old?

1. No 2. Yes 888. Don’t know 999. Refuse to answer

1. How big is your flat? __________ square feets 888 Don’t know 999 Refuse to answer
2. What is your monthly household income (HKD)?

| 1. < 2,000 | 1. 2,000 - 3,999 | 1. 4,000 - 5,999 | 1. 6,000 - 7,999 |
| --- | --- | --- | --- |
| 1. 8,000 - 9,999 | 1. 10,000 - 14,999 | 1. 15,000 - 19,999 | 1. 20,000 - 24,999 |
| 1. 25,000 - 29,999 | 1. 30,000 - 39,999 | 1. 40,000 - 59,999 | 1. ≥ 60,000 |
| 888.Don’t know | 999.Refuse to answer |  |  |

1. Which district do you live in?

| 1. Central & Western | 1. Wan Chai | 1. Eastern | 1. Southern |
| --- | --- | --- | --- |
| 1. YauTsimMong | 1. Sham Shui Po | 1. Kowloon City | 1. Wong Tai Sin |
| 1. Kwun Tong | 1. KwaiTsing | 1. Tsuen Wan | 1. TuenMun |
| 1. Yuen Long | 1. North | 1. Tai Po | 1. Sha Tin |
| 1. Sai Kung (answerQ.73a) | 1. Islands | 888.Don’t know | 999.Refuse to answer |

73a) If you live in Sai Kung District, do you live in Tseung Kwan O? 1. No 2. Yes

1. Do you have a device(s) that has internet access?

a) Personal computer 1. No 2. Yes 888. Don’t know 999. Refuse to answer

b) Tablet 1. No 2. Yes 888. Don’t know 999. Refuse to answer

c) Smart phone 1. No 2. Yes 888. Don’t know 999. Refuse to answer

d) Others__________ 1. No 2. Yes 888. Don’t know 999. Refuse to answer

1. How much time you spend on Internet outside of work daily?

1 .Less than 30 minutes 2. 30 minutes to 2 hours 3. 2 to 4 hours 4. More than 4 hours

888. Don’t know 999. Refuse to answer

1. Does your home have air conditioner?

1. No 2. Yes 888. Don’t know 999. Refuse to answer

1. Does your home have heater?

1. No 2. Yes 888. Don’t know 999. Refuse to answer

78) Are you willing to allow us, the Chinese university of Hong Kong, to call back again to ask your opinion when there are severe infectious disease outbreaks or other emergencies situations in Hong Kong?

1. No (end of the survey) 2. Yes(ask 78a)

888. Don’t know (end of the survey) 999. Refuse to answer (end of the survey)

78a) If agree, please leave your contact number:________________

**== End of Questionnaire. Thank you! ==**

End Time:_________
