# Supplementary material for: Household Preparedness and Preferred Communication Channels in Public Health Emergencies: A Cross-Sectional Survey of Residents in an Asian Developed Urban City
Source: Int J Environ Res Public Health. 2018 Jul 27;15(8):1598. doi: 10.3390/ijerph15081598 (PMC6121418; doi:10.3390/ijerph15081598)
Supplement: Supplementary file 1 [file ijerph-15-01598-s001.zip › IJERPHS1 Table.docx]

**S1 Table. Household preparedness of vulnerable population**

| Characteristics | Good household preparedness | | ^a^COR (95% CI) | P-value |  |
| --- | --- | --- | --- | --- | --- |
|  | Poor | Good |  |  |  |
|  | N (%) | N (%) |  |  |  |
| Age |  |  |  |  |  |
| 15-24 | 55(38.5) | 88(61.5) | 1 |  |  |
| 25-44 | 138(39.7) | 210(60.3) | 0.95(0.64, 1.42) | 0.81 |  |
| 45-64 | 154(42.4) | 209(57.6) | 0.85(0.57, 1.26) | 0.42 |  |
| ≧65 | 69(41.6) | 97(58.4) | 0.88(0.56, 1.39) | 0.58 |  |
| Do you suffer from chronic disease? | | |  |  |  |
| No | 334(41.1) | 479(58.9) | 1 |  |  |
| Yes | 81(39.3) | 125(60.7) | 1.08(0.79, 1.47) | 0.65 |  |
| Do any of your family members suffer from respiratory disease? (e.g. asthma, chronic bronchitis) | | | | |  |
| No | 338(41.3) | 480(58.7) | 1 |  |  |
| Yes | 77(38.3) | 124(61.7) | 1.13(0.83, 0.56) | 0.44 |  |
| Have you caught cold /flu in the past 2 weeks? | | |  |  |  |
| No | 333(40.8) | 484(59.2) | 1 |  |  |
| Yes | 83(40.9) | 120(59.1) | 0.99(0.73, 1.36) | 0.97 |  |
| Are there any member in your household who is under 15 years old or older than 59 years old? | | | | |  |
| No | 155(39.6) | 236(60.4) | 1 |  |  |
| Yes | 260(41.4) | 368(58.6) | 0.93(0.72, 1.20) | 0.58 |  |

^a^COR: Crude odds ratio
