# Supplementary material for: Household Preparedness and Preferred Communication Channels in Public Health Emergencies: A Cross-Sectional Survey of Residents in an Asian Developed Urban City
Source: Int J Environ Res Public Health. 2018 Jul 27;15(8):1598. doi: 10.3390/ijerph15081598 (PMC6121418; doi:10.3390/ijerph15081598)
Supplement: Supplementary file 1 [file ijerph-15-01598-s001.zip › IJERPHS3 Fig.docx]

**Fig. S3. Preferred channels for providing information to officials for surveillance.**
